# Management and outcome of patients with femoral head fractures: the mid-term follow-up with injuries and associated prognostic factors

**DOI:** 10.1186/s12891-023-06317-w

**Published:** 2023-04-20

**Authors:** Sujan Shakya, Jialei Chen, Jiachen Sun, Zhou Xiang

**Affiliations:** Department of Orthopedic Surgery, West China Hospital, Sichuan University, Chengdu, Sichuan 610041 People’s Republic of China

**Keywords:** Avascular necrosis, Femoral head fracture, Heterotopic ossification, Pipkin fracture, Post traumatic osteoarthritis, Surgical approach

## Abstract

**Background:**

Femoral head fractures are rare injuries often associated with poor functional outcomes and complications. The purpose of this study was to evaluate the incidence, treatment methods and approaches, complications, and functional outcomes of femoral head fractures.

**Methods:**

We retrospectively reviewed 50 patients who sustained femoral head fractures between January 2011 and December 2018. There were thirty-seven (74%) males and thirteen (26%) females with a median age of 40 years. According to Pipkin’s classification, there were eighteen (36%) Pipkin I, ten (20%) Pipkin II, eight (16%) Pipkin III, and fourteen (28%) Pipkin IV patients. Treatment methods were categorized into non-operative, operative by open reduction and internal fixation (ORIF), and immediate total hip replacement (THR). The recorded surgical approach consists of an anterior(S-P) approach, posterior(K-L) approach, lateral stab, and combined anterior + lateral stab approach for fixation. The patients were also stratified by the Injury Severity Score (ISS), associated injuries, and, mechanism of injuries. The modified harris hip score (MHHS) was used to evaluate the ongoing complications with the clinical outcome of patients with two years or greater follow-up.

**Results:**

Eight (16%) patients were managed successfully with closed reduction without surgery and thirty-seven (74%) patients required operative reduction and internal fixation (ORIF) of the femoral head and acetabulum, and 5 (10%) patients required immediate THR. Six (12%) patients developed AVN, and four (8%) required a secondary THR. Sixteen patients (33%) developed post-traumatic osteoarthritis (PTOA), eight (16%) developed heterotopic ossification (HO) and six patients (12%) had sciatic nerve injury, none requiring operative treatment. Overall functional results according to MHHS were, excellent in two (4%) patients, good in sixteen (32%) patients, fair in twenty-two (44%) patients, and poor in ten (20%) patients. A statistically significant difference in outcome was observed among four pipkin subtypes.

**Conclusion:**

Femoral head fractures are rare injuries often associated with poor outcomes. In this study, we report the functional outcomes and complications of all treatment approaches for femoral head fracture based on the Pipkin classification. The treatment aim should always be the anatomical reduction of the fragments. This study, adds to the growing literature on femoral head fracture and provides a reference for the clinical treatment to guide patient management.

**Trial registration:**

Our study was approved by the Clinical Research and Biomedical Ethical Committee of West China Hospital, Sichuan University, and was performed in accordance with the Declaration of Helsinki. All participants provided written informed consent to participate in this study.

## Introduction

Femoral head fractures are rare, but severe injuries with potentially significant long-term implications for patients. These fractures are often the result of high-energy trauma due to road vehicle accidents (RTA). The most common mechanism is dashboard injury to the hip and lower extremities, which accounts for approximately 5–15% of posterior hip dislocations [1, 2]. In 1957, Pipkin established a classification system that is most widely used to evaluate femoral head fractures [3–6]. Pipkin categorized these injuries based on the location of the head fracture in relation to the fovea (Ligamentum Teres) and the associated lesions on the femoral neck or acetabulum. Pipkin type I involves the non-weight-bearing part of the femoral head, type II affects the weight-bearing part of the head of the femur, type III may include either or both types I or II with a femoral neck fracture, and type IV involves type I or II associated with an acetabular fracture [7, 8]. Standard treatment strategies for the management of these injuries range from nonoperative treatment to fracture fragment excision or fracture fixation using various surgical approaches and implants [9]. The common surgical approaches in practice include the Kocher–Langenbeck approach, Smith–Petersen approach, Hueter approach, Watson–Jones approach, the greater trochanter osteotomy approach, and the Ganz approach [10]. However, the optimal management strategy for femoral head fractures remains unclear. Closed non-surgical treatment can be the approach for Pipkin type I and II fractures; however, there is debate as to whether the treatment should be operative or non-operative [11]. There is still no consensus on the management of injuries, whether to treat these fractures operatively or non-operatively, whether to fix or excise the head fragment by open reduction or arthroscopically assisted or which surgical approach to use [4, 12–14]. THR is an option that is often recommended in lesions involving femoral head fracture in elderly patients or severely damaged femoral head associated with acetabulum components [15].

Regardless of the type of treatment, long-term complications, such as avascular necrosis (AVN), post-traumatic osteoarthritis (PTA), sciatic nerve palsy, and heterotopic ossification (HO), may lead to unfavorable and potentially varying degrees of disability in patient outcomes [1, 16–18]. Early recognition and prompt treatment are important for the successful management of patients with femoral head fractures and hip dislocations [16]. However, the outcomes of patients with femoral head fractures remain poor owing to the lack of absolute recommendations and indications for fracture management. Few studies have reported on the outcome and management of femoral head fractures; however, there are limitations because of the inconsistent fracture classification scheme with prognostic significance, multiple treatment approaches, small patient size, insufficient length of follow-up, and use of non-validated outcome instruments [7, 18, 19].

The aim of this study was: i) to investigate the management of femoral head fractures which been managed non-surgically or surgically; ii) to evaluate postoperative complications and prognostic factors, and iii) to analyze the functional outcome using modified Harris Hip Score to provide a reference for the clinical treatment.

## Materials and methods

**Fig. 1:**
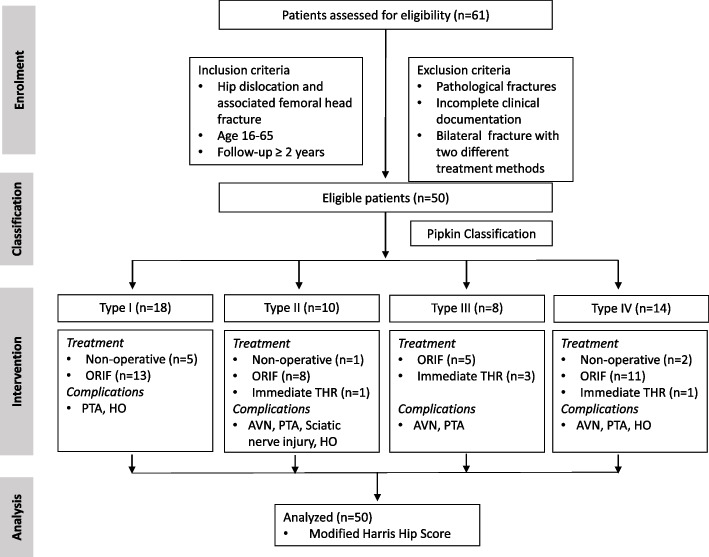
Flow diagram showing femoral head fracture patients’ recruitment, clinical intervention and analysis

Between January 2011- December 2018, sixty-one femoral head fractures treated in a Level I trauma center were retrospectively followed with data recorded contemporaneously in an IRB-approved registry. Inclusion criteria were as follows: i) age 16–65, ii) follow-up of two years or greater after the femoral head injury operatively or non-operatively, and (iii) acute traumatic femoral head fracture with at least an available plain anteroposterior (AP) radiograph of the affected hip. Patients were excluded if they presented with pathological or non-acute fractures, or dislocation of the femoral head. Incomplete radiographic evaluation or unavailable clinical documentation (Fig. 1). In addition, one patient who had undergone both ORIF and THR was excluded. The data collected from each patient included demographics, fracture type, presence of associated injury, injury severity score (ISS), mechanism of injury, operation time, intensive care unit care, operation time, intraoperative blood loss, clinical outcomes, and mortality. Patients were classified according to the Pipkin classification system [7, 20, 21].

### Treatment and management

The treatment approach and timing for recovery for each patient were dependent based on the fracture pattern and associated injuries. The need for operative intervention was determined based on general guidelines: hip instability, large intra-articular fragments greater than 2 mm, and bone or cartilaginous fragments in the joint space [17, 22]. In cases of open reduction and internal fixation, the anterior Smith-Peterson (S-P) and posterior Kocher-Langenbeck (K-L) approaches were used depending on the type and location of the fractured fragments. The choice of fixator was based on the size and location of the fracture and the surgeon’s preference. Reconstruction plates and interfragmentary Herbert screws were used to obtain stable anatomical fixation in the acetabulum and femoral head, respectively. For patients undergoing operative intervention, the operation time, intraoperative blood loss, surgical approach, and type of fixation were recorded. In our study, reduction was performed within six hours of fracture dislocation. If the patients underwent nonoperative management, skeletal traction was continued for at least 6 weeks and was deemed to have stable fracture patterns. Postoperatively, the patients were encouraged to perform isometric exercises for the quadriceps and lower limb muscles. Simultaneously, patients with THR were asked to undergo early postoperative mobilization.

### Evaluation of clinical outcome

Patient outcomes and complications were determined based on a review of clinical and radiographic results from the most recent follow-up. The median follow-up period was 36 months (range: 24–84 months). The Brooker classification system was used to evaluate HO formation. Patients with HO did not receive any prophylactic radiation or NSAIDs other than analgesic medications for acute pain management. The presence of post-traumatic osteoarthritis, osteonecrosis of the head, and heterotopic ossification changes was assessed using functional scores and radiological changes by experienced orthopedic surgeons during each follow-up visit. Other complications, such as postoperative infection, deep venous thrombosis (DVT), and sciatic nerve injury were also documented. Sciatic nerve injury and peroneal division were diagnosed during the physical examination. Functional recovery was evaluated according to MHHS criteria at the latest clinical follow-up.

### Modified harris hip score (MHHS)

The MHHS is a patient-based questionnaire that is a relatively simple process to assess the pain, functional status, and functional activities of the hip. It is a tool used to calculate the score of the functional outcome based on the physical examination components by saving the time and energy of the clinical practitioner. In the absence of a patient, questionnaires can be completed by phone or through correspondence. The thresholds for outcome classification using the MHHS were as follows: < 70 (poor result), 70–79 (fair result), 80–89 (good result), and > 90 (excellent result). The MHHS is a surgeon-derived outcome measure that contains eight items representing the main aspects of pain, functional gait, and functional activities [23–25].

### Statistical analysis

Statistical analysis was performed with GraphPad Prism 8.0 software (GraphPad Software, Inc., CA, USA). For quantitative variables, the data were expressed as mean ± standard deviation (SD). The sample size for each variable is included in the figure legends. *P*-values were calculated using one-way ANOVA *P-*value of < 0.05 was considered to be significant.

## Results

### Patients and injury characteristics (Demographics)

Sixty-one patients were assessed for eligibility, as shown in the flow diagram (Fig. 1). Fifty patients with femoral head fractures met the eligibility criteria and were included in this study. There were 37 men and 13 women, with a median age of 40 years at the time of injury (range 16–65). Thirty-one patients sustained fractures from a motor vehicle accident, 14 from a fall, two from sports, two from a bike, and one from a workplace-related accident. There were eighteen Pipkin I fracture, ten Pipkin II, eight Pipkin IIII, and fourteen Pipkin IV fracture according to Pipkin classification. Car accidents were the most common injury mechanism (31/50), followed by fall injuries (14/50). Patients were stratified by ISS into four groups: (a) mild (ISS, 9), (b) mild-to-moderate (ISS, 10–15), (c) moderate-to-severe (ISS, 16–25), and (d) severe (ISS, > 26). There were 20 mild, 11 mild-to-moderate, 17 moderate-to-severe, and 2 severe ISS patients. There were 20 patients with orthopedic cases of femoral head fractures and 30 with orthopedic cases associated with polytrauma. The patient demographics, classifications, and associated injuries are listed in Table 1.

**Table 1:** Table showing demographic, classification, associated injuries and mechanism of injuries related to femoral head fractures

Parameters	Number of patients (%)
Number of Patients	50
Gender	
Male	37 (74%)
Female	13 (26%)
Classification	
Pipkin I	18 (36%)
Pipkin II	10 (20%)
Pipkin III	8 (16%)
Pipkin IV	14 (28%)
Associated injuries	
Knee contusion	15 (29%)
Patella fracture	12 (23%)
Extremities fracture	13 (25%)
Ribs fracture	16 (31%)
Lumbar transverse process fracture	10 (20%)
Pubic rami fracture	5 (10%)
Chest contusion	7 (14%)
Sciatic nerve damage	6 (12%)
Brain contusion	6 (12%)
Clavicle fracture	1 (2%)
Hemorrhagic anemia	3 (6%)
20 isolated pipkin fracture out of 51	40%
30 polytraumatic patients with associated injuries	60%
Mechanism of injury	
Car accident	31(62%)
Fall injury	14 (28%)
Sports injury	2 (4%)
Bike accident	2 (4%)
Workplace accident	1 (2%)

## Management

**Fig. 2:**
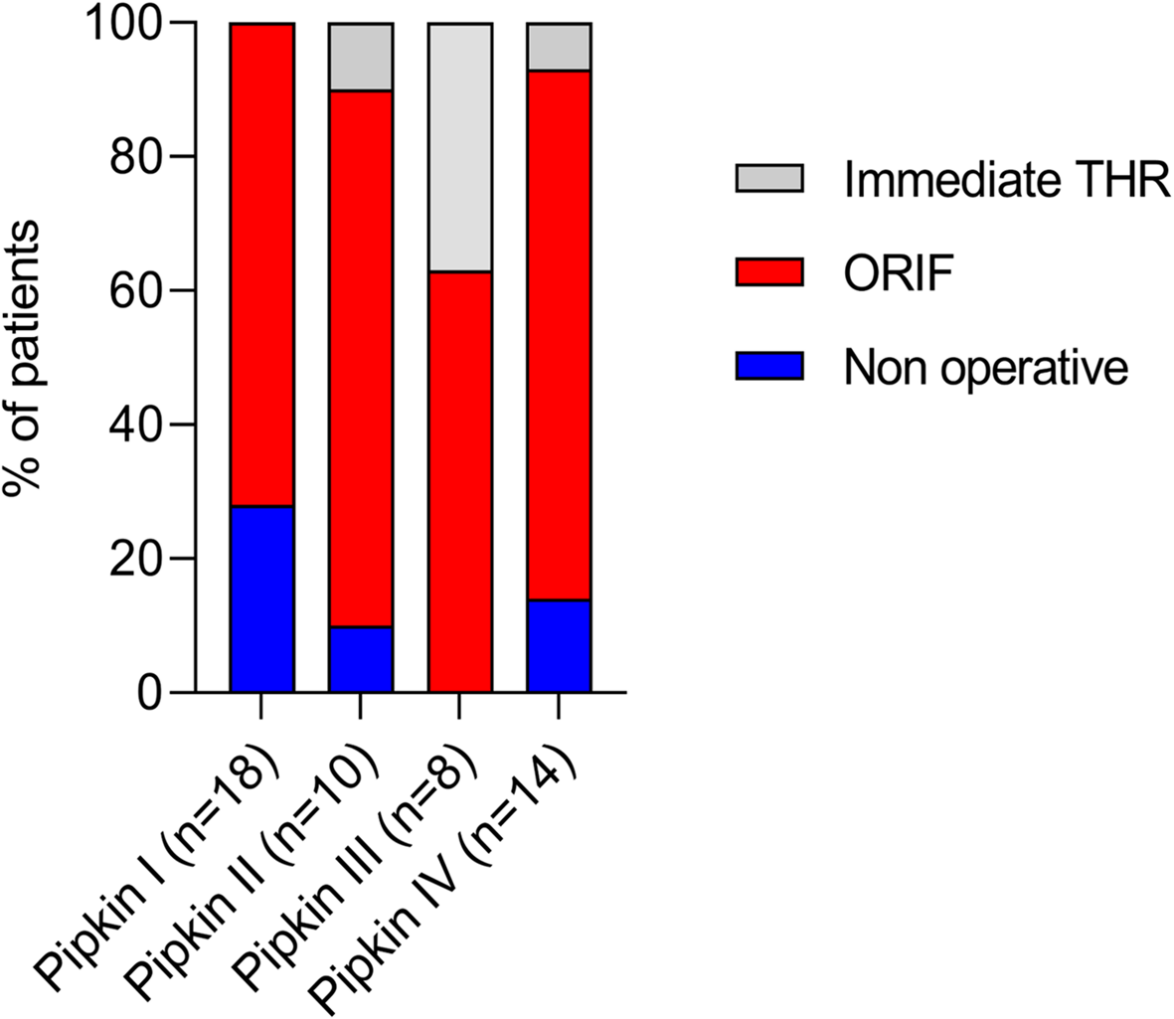
Relative distribution of femoral head fracture in each Pipkin subtype based on different treatment variables

Of the 50 patients, thirty-seven (74%) were managed with ORIF for the femoral head and acetabulum, five (10%) underwent immediate THR, and eight (16%) were treated nonoperatively. The overall treatment, in relation to the Pipkin classification, is shown in Table 2. Non-operative intervention was mainly rendered to patients with Pipkin Type I (28%) fractures, while ORIF was performed mostly for Pipkin type II (80%) and type IV (79%) fractures. Notably, the majority (60%) of patients who had immediate THR were within the Pipkin type III subgroup. Two patients with Pipkin III fractures and one patient with Pipkin IV fractures, who were treated surgically using ORIF, required an eventful conversion to secondary THR. In addition, one patient with Pipkin II fractures who was treated non-operatively required secondary conversion to THR. Next, we examined different treatment variables, such as non-operative, ORIF, immediate THR, and combined ORIF/THR, for each Pipkin subtype (Fig. 2).

**Table 2:** Table showing treatments, complications and outcomes of femoral head injury

	No. of patients (%)
Treatments	
Nonoperative	8 (16%)
Operative	
ORIF	37 (74%)
Immediate THR	5 (10%)
Complication	
AVN	6 (12%)
PTA	16 (32%)
HO	8 (16%)
Sciatic nerve injury	6(12%)
Modified Harris Score	
Excellent	2 (4%)
Good	16 (32%)
Fair	22 (44%)
Poor	10 (20%)
Secondary THR	4 (8%)

*ORIF* open reduction and internal fixation, *AVN* avascular necrosis, *PTA* post traumatic arthritis, *HO* Heterotopic ossification, *THR* Total Hip Replacement

Regarding the surgical approaches, the anterior (Smith—Peterson) approach was used in 18 patients while the posterior (Kocher-Langenbeck) approach was used in 19 patients. The posterior K-L approach was used for immediate or secondary THR. Three patients were treated with a lateral stab approach. Two patients were treated using combined anterior and lateral approaches. Cannulated screws were used in the lateral approach that is percutaneously inserted into the femoral neck by a stab incision. Three patients who underwent ORIF using a lateral approach and two patients who underwent ORIF using the posterior approach developed AVN accompanied by posttraumatic arthritis and required eventful conversion to a secondary THR. Patients treated nonoperatively were believed to be too fragile for surgery due to the severity of their associated injury and medical comorbidities or were deemed to have stable fracture patterns, and therefore treated with traction.

**Fig. 3:**
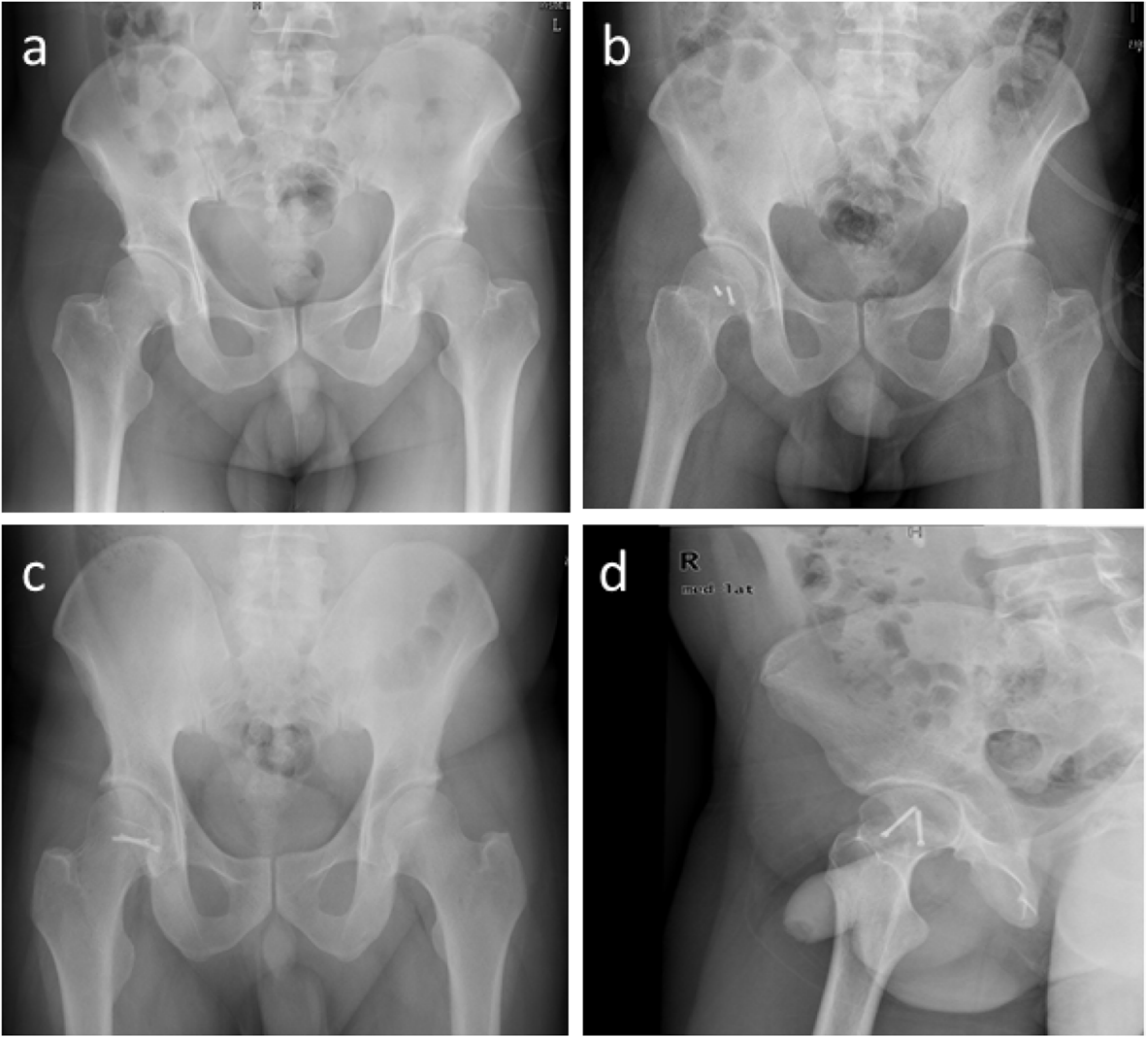
**a** A 39 -year-old man with Pipkin type I right femoral head fracture **b** Anteroposterior (AP) radiographs showing anatomical reduction of the femoral head using anterior approach under direct vision. **c** AP and **d** Lateral radiograph of the hip during 3rd-year follow-up after internal fixation

**Fig. 4:**
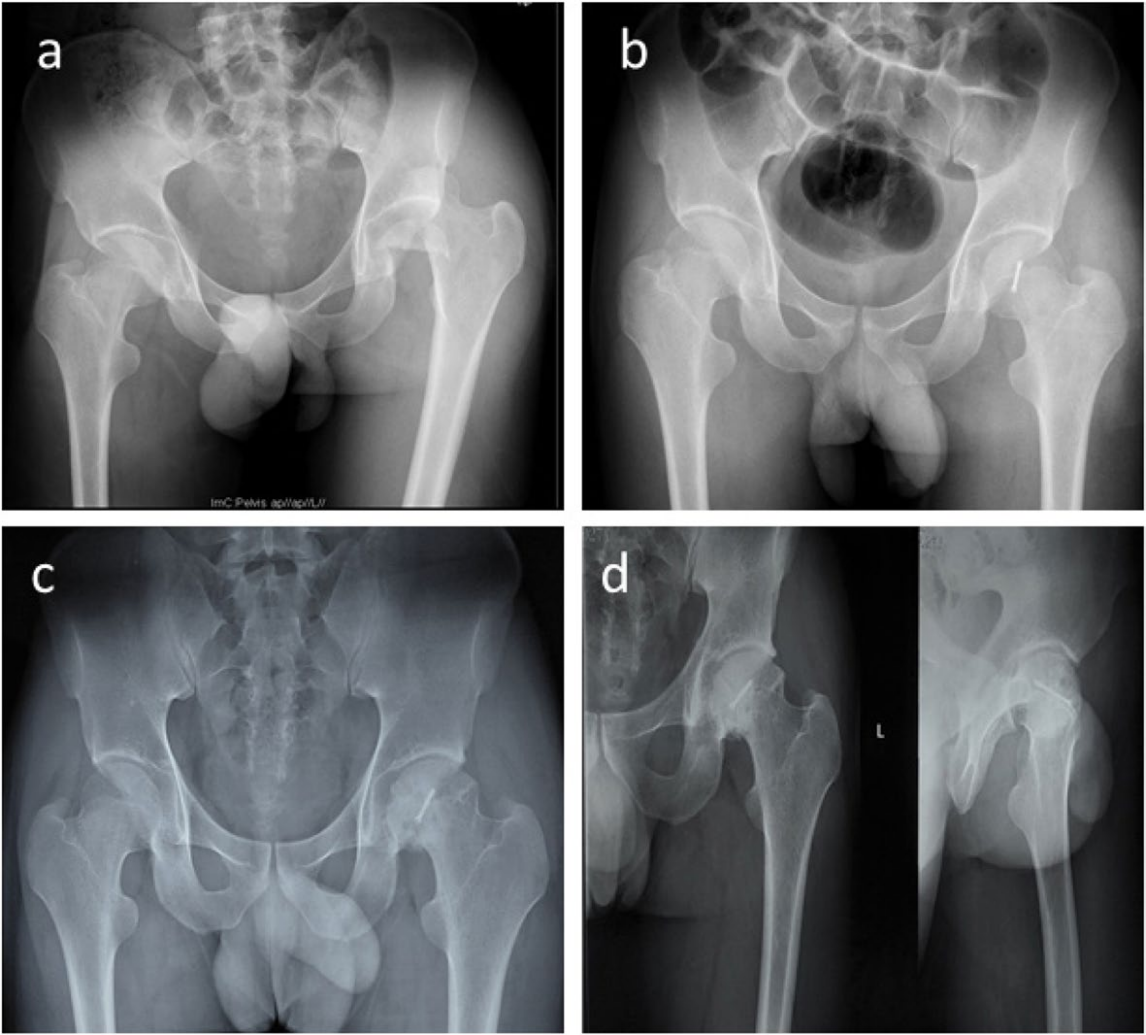
**a** Anteroposterior pelvic radiographs showing a 21-year-old male with left Pipkin II fracture-dislocation. **b** Post-operative X-ray films showing anatomical reduction of the femoral head with one Herbert screw via an anterior approach. **c** AP radiograph of 4th-year follow-up. **d** AP and Lateral radiograph of 7th-year follow-up.  There is evidence of post-traumatic osteoarthritis, including some loss of joint space, but there are no indications of AVN or heterotopic ossification

**Fig. 5:**
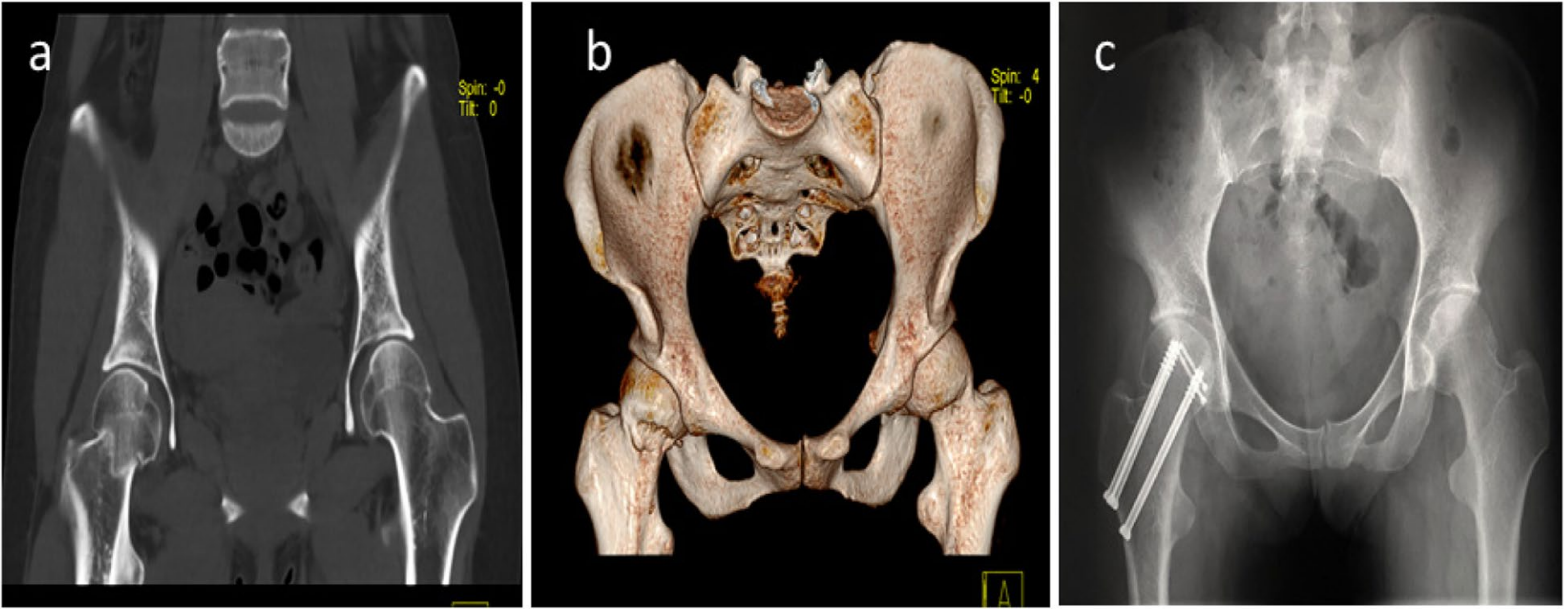
A 27-year female sustained injury after motor vehicle accident. **a** Coronal view **b** 3D CT demonstrated right sided Pipkin III femoral head and neck fracture. **c** The fracture was anatomically reduced with herbert screw via anterior approach and femoral neck was corrected with three cannulated screw utilizing lateral stab approach

The association between operative approaches with Pipkin subtypes was also examined. An anterior approach was mainly used for patients with Pipkin I and Pipkin II fracture (Figs. 3 and 4), while the majority of patients with Pipkin III and IV fractures were treated using the posterior approach. Combined anterior and lateral stab approaches were used in the Pipkin III fracture (Fig. 5).

## Complications

The overall incidence of mid-term complications (median follow-up 36 months) was evaluated. Six patients developed AVN for an overall incidence of 12%. Four (8%) of these patients required eventful conversion to a secondary THR. Sixteen patients (32%) had radiographic criteria of PTOA at their latest clinical follow-up. Two patients (4%) had iatrogenic sciatic nerve injuries. There was one patient who was diagnosed with post-operative superficial infection and one with DVT in the lower limb. Both of these patients were improved by receiving medication and without surgical management. Eight patients (16%) developed HO. This was graded as Brooker I in all eight patients. None of these patients required operative intervention.

We next examined the relationship between the preferred surgical approaches and complications encountered, mainly AVN, PTA, sciatic nerve palsy, and HO (Table 3). Odds ratio analysis revealed that the incidence of HO (all Brooker I) was 1.7 times higher after the posterior approach than after the anterior approach. However, this difference was not statistically significant. There was not incidence of HO when the lateral approach was used. Similarly, the post-traumatic arthritis incidence was estimated when a lateral approach was used rather than a posterior or an anterior approach, while 1.6 times higher after a posterior approach in comparison to the anterior approach. However, the difference was not statistically significant. Of the 19 patients, two patients who were treated using the posterior approach developed AVN. Interestingly, all patients who underwent the lateral approach developed AVN, whereas there was no incidence of AVN when an anterior approach was used. Similarly, none of the patients treated using the combined surgical approach experienced major late complications.

**Table 3:** Table showing treatments, complications and outcome of femoral head injury according on pipkin classification

	n = 50	Pipkin I (*n* = 18)	Pipkin II (*n* = 10)	Pipkin III (*n* = 8)	Pipkin IV (*n* = 14)
Treatments					
Non-operative	8 (16%)	5 (28%)	1 (10%)	0 (0%)	2 (14%)
Operative					
ORIF	37 (74%)	13 (72%)	8 (80%)	5 (63%)	11 (79%)
Immediate THR	5 (10%)	0 (0%)	1 (10%)	3 (37%)	1 (7%)
Complications					
AVN	6 (14%)	0 (0%)	1 (10%)	3 (38%)	2 (14%)
PTA	16 (33%)	4 (22%)	3 (20%)	3 (38%)	6 (43%)
HO	8 (16%)	2 (11%)	3 (30%)	0 (0%)	3 (21%)
Sciatic nerve injury	6(12%)	2 (0%)	0 (0%)	2 (25%)	2 (14%)
Modified Harris Score					
Excellent	2 (4%)	2 (11%)	0 (0%)	0 (0%)	0 (0%)
Good	16 (32%)	9 (50%)	5 (50%)	0 (0%)	2 (14%)
Fair	22 (44%)	5 (28%)	4 (40%)	5 (64%)	8 (57%)
Poor	10 (20%)	2 (11%)	1 (10%)	3 (36%)	4 (29%)
Secondary THR	4 (8%)	0 (0%)	1 (10%)	2 (25%)	1 (7%)

*ORIF* open reduction and internal fixation, *AVN* avascular necrosis, *PTA* post traumatic arthritis, *HO* Heterotopic ossification, *THR* Total Hip Replacement, *N* number of patients

## Functional outcome

Clinical and radiographic data were reviewed for all patients at their latest clinical follow-up and collected according to the MHHS. According to the MHHS criteria, the overall clinical results were excellent in 2 (4%) patients, good in 16 (32%), fair in 22 (44%), and poor in 10 (20%). Four patients with poor outcomes developed AVN and PTA, and underwent eventful conversion to secondary THR.

**Fig. 6:**
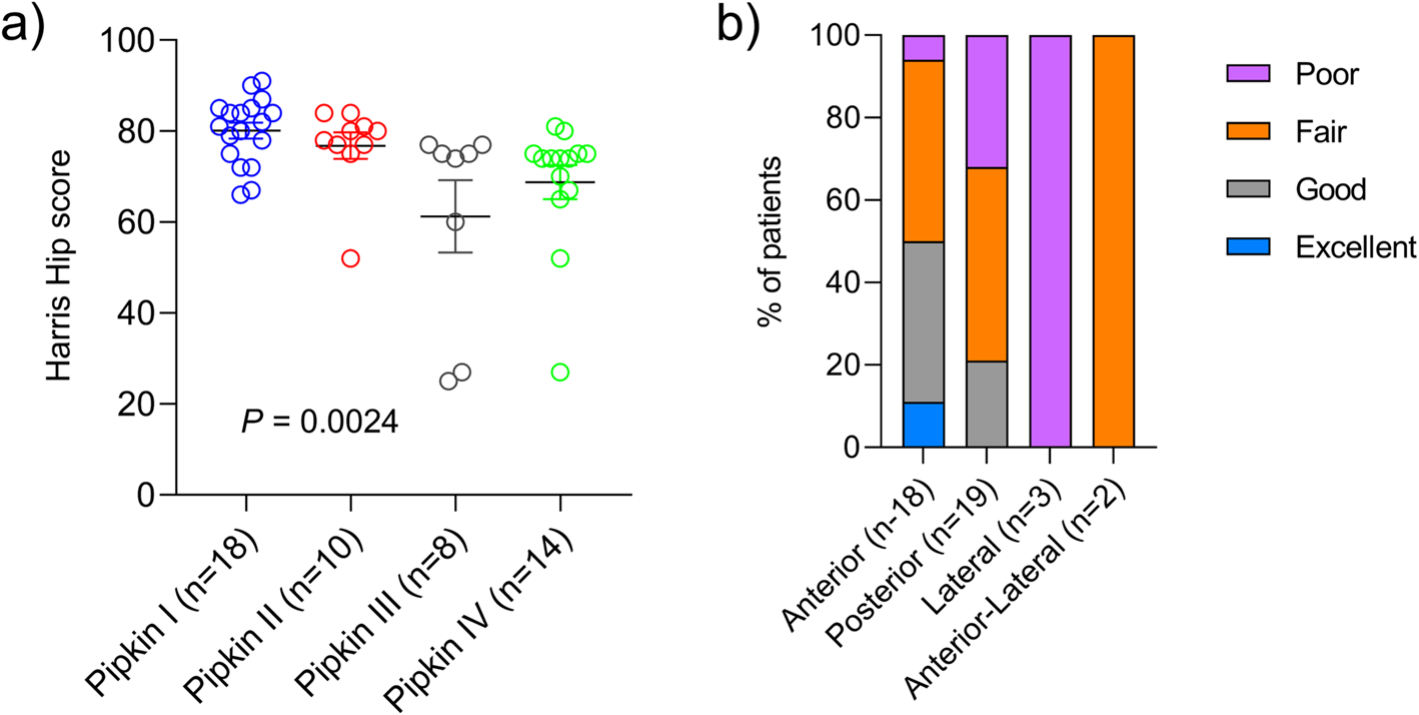
**a** Harris hip score measuring functional outcome in relation to different surgical approaches. Data presented as mean ± SD. P-values were calculated using one-way NOVA (analysis of variance). *P* < 0.05 considered as statistically significant. **b** Relative distribution of femoral head fracture patients in four Pipkin subtypes based on functional outcome

Next, we investigated the relationship between the results of the MHHS and Pipkin Classification (Table 4). The overall outcome interpretation was further subdivided based on the Pipkin classification. Majority of the patient with Pipkin I fracture showed excellent functional outcomes, while the outcome of patients with Pipkin III and Pipkin IV fractures was relatively poor compared to Pipkin I and Pipkin II. Statistical analysis revealed a significant (p = 0.0024) difference in outcome among pipkin subtypes, indicating a variance of functional outcome value according to the Pipkin classification in femoral head fractures (Fig. 6a). However, this may also be due to the potential confounding effects of the different treatment strategies. We further examined the relationship between outcome, according to the Harris Hip score, and each treatment variable (non-operative, ORIF, Immediate THR) (Table 5). For the non-operative group, the results were good in five (62.5%), fair in two (25%), and poor in one (12.5%). Among the surgically treated patients, the outcomes were excellent in 2(4.7%), good in 11(26.1%), fair in 20 (35.7%), and poor in 10(21.4%). The outcomes of 5 (100%) patients who underwent primary intention THR were fair. Because of the small number of patients in our cohort, we were unable to examine the influence of the confounding effect of the treatment strategy on functional outcomes. Furthermore, the relationship between the functional outcomes and operative approaches was examined. The outcomes of patients treated using the anterior approach were excellent in 11%, good in 39%, fair in 44%, and poor in 6%. While the outcome was mostly fair in nine patients (47%), four (21%) were good, and six (32%) were poor using the posterior approach. The relationship between functional outcomes and surgical approaches used was also examined (Table 6). While there was no difference in outcome between the anterior and posterior approaches, the majority (79%) of patients treated using the posterior approach had a poor or fair outcome. None of the patients treated using the lateral approach showed better outcomes (excellent or good) (Fig. 6b).

**Table 4:** Table showing complications in relation to the surgical approaches

Complications	Anterior (S-P) (*n* = 18)	Posterior (K-L) (*n* = 19)	Lateral (*n* = 3)	Anterior + Lateral (*n* = 2)	Total (*n* = 42)
AVN		2	3		5 (12%)
PTA	4	6	3		13 (31%)
HO	3	5			8 (19)
Sciatic nerve injury		2			2(5%)

*AVN* avascular necrosis, *PTA* post traumatic arthritis, *HO* Heterotopic ossification, *S-P* Smith-Peterson, *K-L* Kocher-Langenbeck, *n* number of patients

**Table 5:** Table showing outcome in relation to the treatment variables

Complications	Non-operative (*n* = 8)	ORIF (n = 37)	THR (*n* = 5)	Total (*n* = 50)
Excellent	0	2 (5.4%)		2 (4%)
Good	5 (62.5%)	11 (29.8%)		16 (32%)
Fair	2 (25%)	15 (40.5%)	5(100%)	22 (44%)
Poor	1 (12.5%)	9 (24.3%)		10 (20%)

*n* number of patients

**Table 6:** Table showing outcome in relation to the surgical approaches

Complications	Anterior (S-P) (*n* = 18)	Posterior (K-L) (*n* = 19)	Lateral (*n* = 3)	Anterior + Lateral (*n* = 2)	Total (*n* = 42)
Excellent	2(11.1%)	0			2(4.8%)
Good	7(38.9%)	4(21%)			11(26.2%)
Fair	8(44.4%)	9(47.4%)		2(100%)	19(45.2%)
Poor	1(5.6%)	6(31.6%)	3(100%)		10(28.8%)

*n* number of patients

## Discussion

Femoral head fracture is a rare injury that typically occurs as a result of traumatic posterior dislocation of the hip joint [16, 26–28]. Early diagnosis and prompt concentric reduction are essential for the successful management of these fractures [21]. However, owing to a lack of established consensus on the diagnosis and treatment of femoral head fractures and the limited number of cases reported in the literature, the prognosis of these injuries remains uncertain.

In this retrospective study, we evaluated the management, complications, and outcomes of patients with femoral head fractures. We used the MHHS to evaluate the functional outcomes. Our study found an overall outcome of excellent in two patients, good in 16 patients, fair in twenty-two patients, and poor in 11 patients. The association between functional outcomes, treatment approaches, and complications was further investigated based on the Pipkin Classification.

According to the Pipkin classification [3–5], a relative increase in poor outcomes from Pipkin 1 to 4 (11% to 29% respectively) was noted. Our study also indicated similar classification-wise outcomes, supported by statistical significance. While these observations were in a small cohort of patients, they do suggest the importance of Pipkin classification in predicting less favorable outcomes with an associated femoral head fracture.

### The particularity of femoral head fractures and prognostic factors

Femoral head fracture with hip dislocation is a true emergency of orthopedic trauma. Long-term fracture and dislocation of the femoral head damage the blood supply to the femoral head, leading to subsequent avascular necrosis of the femoral head [18]. In addition, complications such as traumatic arthritis may develop due to poor reduction of fractures in the weight-bearing area of the articular surface [29]. Therefore, timely diagnosis and prompt reduction of the associated hip dislocation should be performed to prevent further damage to the peripheral vessels and improve outcomes. Treatment measures were either operative or nonoperative. The treatment approach and timing of recovery for each patient were dependent on the fracture pattern and associated injuries. Using skeletal traction [9, 30–32], which is frequently used for the initial management of femoral head fractures, 16% of cases in our study were managed non-operatively to decrease the risk of chondrolysis. The criteria for non-operative intervention were determined based on anatomic reduction of hip dislocation and femoral head fracture, intra-articular fragment displacement of less than 1 cm, absence of bone or cartilaginous fragment in the joint space, and hip stability. Those fractures that did not meet such criteria were treated operatively [8, 16]. Operative measures included fracture fixation using ORIF or THR. Operative management is generally preferred when the fracture is severe and extends superiorly to the fovea. In our study, ORIF was mainly rendered to Pipkin II (80%) and Pipkin IV (79%) fracture, while THR was performed mostly within Pipkin III fracture (37.5%).

The long-term follow-up analysis after operative (ORIF) or non-operative treatment regimens on Pipkin I injuries demonstrated that the best results (80% excellent or good) were accomplished. Although a statistical difference was not found (*P* = 0.59), the non-operative intervention seems to result in a better outcome than an operative intervention. Several studies support this non-operative management of Pipkin I fracture and controversies remain regarding the surgical management of these fractures [33–36]. The fact that only 5 cases were managed non-operative. Thus, we do not make an absolute recommendation in favor of non-operative when dealing with Pipkin 1. However, when the head fractures are less than 1 mm, absence of loose bodies in the joint space, stable hip joint with good relation of the head with the glenoid [37], non-operative intervention may be an adequate intervention. Pipkin II fracture involves a larger portion of the weight-bearing femoral head surface and is a more challenging injury [33]. The majority (80%) of these fractures were operated with internal fixation of the fragment. This is in line with current principles of managing Pipkin II fractures with anatomical reduction and surgical fixation [11, 18, 33].

Pipkin Type III fracture is the least frequent type that involves dual insult to the femoral head and neck. All eight of our patients with Pipkin III injuries underwent operative intervention using ORIF and/or THR, while none of the patients demonstrated the best results (excellent or good). Although the treatment options for Pipkin III fractures range from open reduction and rigid fixation to arthroplasty, the outcome is highly dependent on age, delay in surgery, and degree of comminution. Generally, young patients with Pipkin III fractures should aim to preserve the joints, while THR may be a reasonable option for the elderly [6, 38]. In our study, two (out of five) patients with Pipkin III fractures who underwent fixation of the fragment required conversion to secondary THR. This trend supports the opinion of published literature that postulates Pipkin III fracture as a predictive of secondary THR in femoral head fracture [7, 39].

Pipkin IV injuries lead to the worst outcome as they involve both the femoral head and the acetabulum. A majority of our patients with Pipkin IV injuries were treated with ORIF, however, there was no significant improvement in outcome among different treatment methods. One of the particular characteristics of this injury group is that, despite the type of intervention used, it is often challenging to address whether the approach should be directed to the acetabulum, femoral head, or both. These fractures require anatomical reduction and internal fixation of the femoral head and acetabulum lesions with attention toward restorations of hip congruency and hip stability.

### Femoral head fracture and significance of surgical approach

Despite advances in several surgical approaches for femoral head fracture management, controversy exists concerning the choice of optimal surgical treatment. The anterior S-P approach offers good exposure and easier access to the fractured head; thus, it is more suitable for the treatment of Pipkin I and II femoral head fractures [40]. Such an anterior approach can significantly reduce blood loss and operation time, and therefore reduce the incidence of avascular necrosis of the femoral head, compared to the posterior K-L approach. However, the often-quoted disadvantage of the anterior-based approaches has been the association with increased heterotopic ossification [4, 13, 17, 41]. Similarly, this approach has also been linked to further damage to any residual anterior blood supply to the femoral head although, the anatomical studies do not support this theory [42, 43]. The posterior-based approach can provide direct visualization of the acetabular fracture and an opportunity for simultaneous repair of the femoral head and acetabular fracture as seen in Pipkin type IV injuries.

We believe that this is the first study to generate a new concept and strategy to use the combined surgical window approach for pipkin III. Here, reduction and fixation of the femoral head was achieved using Herbert screws using the anterior S-P approach. In the same window, reduction and preliminary fixation of the femoral neck were achieved, and a separate lateral stab approach was utilized for cannulated screw implant insertion. This strategic method has been proven to have good functional outcomes and low complication rates in patients to improve the prognosis of Pipkin III. However, the prevalence of Pipkin III was low, and our study included fewer patients. A larger study can be conducted using this strategy. A recent study also aimed to explore the efficacy of the direct anterior and posterior approach in Pipkin IV femoral head fracture leading to a favorable prognosis while not increasing the incidence of complications [44, 45].

Correlation analysis showed no statistical difference (p > 0.05) in outcome between the anterior and posterior approaches. Although it should be noted that irrespective of our findings, the choice of surgical approach and outcome is frequently determined by the fracture pattern and the overall injury severity characteristics.

### Factors regarding complications and outcomes

Regarding major complications, our findings suggest that the likelihood of AVN is higher when the lateral stab approach is used. This could be due to the severity of Pipkin III injuries and confounding factors such as displaced femoral neck fracture, damage to vascular structures, and inadequate reduction that mostly leads to subsequent AVN despite surgical approaches. AVN is one of the main long-term complications secondary to iatrogenic insult or damage during the initial injury [22]. The clinical symptoms of AVN may present early (from 6 weeks) or late (several years following injury) with collapse of the femoral head accompanied by PTA [46].

In our study, all the patients who developed AVN showed poor functional outcomes. Two patients who underwent a posterior approach for ORIF developed an AVN. It is important to note that our mean follow-up time may be too short to capture all patients who developed clinical symptoms of AVN. Thus, longer follow-up periods are required for a detailed analysis of the incidence of AVN.

Currently, in clinical practice, there is no routine examination to assess the blood flow of the femoral head after a Pipkin fracture, except for MRI, for the diagnosis of AVN. Recent studies have quantified the blood flow and perfusion status of the femoral head after hip fracture using single-photon emission computed tomography (SPECT) with computed tomography (CT) to evaluate the blood flow status in the bone while precisely localizing the necrotic site [47–49]. We recommend initiating the trend to judge the blood flow status with axillary examination with SPECT or PET-CT to predict osteonecrosis of the femoral head even after implant insertion after surgical or non-surgical management of Pipkin fractures.

Similarly, HO is one of the most common complications after operative fixation, with an incident associated with the anterior surgical approach [13, 18, 26]. In our study, odds ratio analysis demonstrated a trend to a higher incidence of HO (all Brooker stages) after the posterior approach relative to the anterior one, which was statistically not significant. Although it is unclear, this result could be implicated due to extensive surgical dissection of gluteal muscles during fixation [50]. However, only eight (16%) of our patients who developed HO, all with a Brooker grade I, had no impact on the final functional outcome. Post-traumatic osteoarthritis is another common complication of femoral head fracture management and its incidence is directly related to the severity of the initial injury [28]. A higher incidence of PTA was found in the case of a posterior or lateral approach respectively versus an anterior approach. This finding, however, could be attributed to the fact that majority of the patients who developed PTA had fracture that belonged to Pipkin type III (37.5%) and IV (43%) category.

Overall, our study adhered to the intra-articular anatomical reduction of Pipkin fractures by operative management. Non-operative intervention may be adequate for Pipkin I fractures and should be recommended only after acceptable evaluation of fracture reduction, articular congruency, hip stability, and the absence of loose fragments in joint space using modern imaging techniques. According to the literature, the majority of the blood supply in the hip comes from the posterior MFCA deep branch, which gets endangered with the posterior approach [45, 51]. Therefore, in order to preserve major vessels, the anterior surgical approach has provided promising results with a lower incidence of major complications, making it probably the best approach for the operative management of Pipkin I and II fractures. The incidence of HO is also high in the posterior K-L approach. Regardless of rigid and anatomical fixation, the degree of trauma with pipkin III or IV creates complexity in physioanatomical healing and poor functional outcomes. Hence, we advocate THA for cases with comminuted fracture block or severe collapse of the cartilage on the load-bearing surface of the femoral head.

This study has several limitations. First, this study was small in size and conducted at a single center. Second, this study enrolled a small number of patients treated using a different approach. Third, the statistical power was low owing to the lack of a higher number of enrolled patents, different approaches, and management. The relatively short follow-up duration was also a limitation of this study because it might have been insufficient to assess post-traumatic osteoarthritis. Therefore, it is important to conduct a large prospective study using validated outcome scores to develop fracture classifications and operative approaches.

## Conclusion

Our experience concludes that femoral head fractures are rare injuries often associated with poor outcomes. Despite appropriate surgical treatment and approach, the risk factors for complications are high, such as AVN, PTA, and HO, which directly correlate with the final functional outcome. The prevalence of good results decreased from types I to IV. This study adds to the growing literature on femoral head fractures and provides a reference for clinical treatments to guide patient management.

## Data Availability

The authors confirm that the data supporting the conclusions of this study is available within the article.

## Abbreviations

ORIF: Operative Reduction and Internal Fixation
THA: Total Hip Arthroplasty
AVN: Avascular Necrosis
PTOA: Post-Traumatic Osteoarthritis
HO: Heterotopic Ossification
S-P: Smith-Peterson
K-L: Kocher-Langenbeck
MHHS: Modified Harris Hip Score
DVT: Deep Venous Thrombosis
SEM: Means ± Standard Error of the Mean
AP: Anteroposterior
CT: Computerized Tomography
ISS: Injury Severity Score
